# Hepatitis C Clearance by Direct-Acting Antivirals Impacts Glucose and Lipid Homeostasis

**DOI:** 10.3390/jcm9092702

**Published:** 2020-08-21

**Authors:** Christiana Graf, Tania Welzel, Dimitra Bogdanou, Johannes Vermehren, Anita Beckel, Jörg Bojunga, Mireen Friedrich-Rust, Julia Dietz, Alica Kubesch, Antonia Mondorf, Sarah Fischer, Thomas Lutz, Philipp Stoffers, Eva Herrmann, Thierry Poynard, Stefan Zeuzem, Georg Dultz, Ulrike Mihm

**Affiliations:** 1 Department of Internal Medicine, University Hospital Frankfurt, 60596 Frankfurt, Germany; tmwelzel@web.de (T.W.); bogdanou@endomedica.gr (D.B.); jvermehren@gmx.de (J.V.); anita.beckel@kgu.de (A.B.); Joerg.Bojunga@kgu.de (J.B.); mireen.friedrich-rust@kgu.de (M.F.-R.); julia.dietz@kgu.de (J.D.); alica.kubesch@kgu.de (A.K.); antonia.mondorf@kgu.de (A.M.); philipp.stoffers@kgu.de (P.S.); stefan.zeuzem@kgu.de (S.Z.); Georg.Dultz@kgu.de (G.D.); ulrike.mihm@kgu.de (U.M.); 2 Infektiologikum, Center for Infectious Diseases, 60596 Frankfurt, Germany; fischer@infektiologikum.de (S.F.); lutz@infektiologikum.de (T.L.); 3 Institute of Biostatistics and Mathematical Modeling, Goethe University Frankfurt, 60596 Frankfurt, Germany; herrmann@med.uni-frankfurt.de; 4 BioPredictive, 75007 Paris, France; thierry@poynard.com

**Keywords:** hepatitis C virus, hepatic fibrosis, insulin resistance, direct-acting antivirals

## Abstract

Background: Chronic hepatitis C virus (HCV) infections are causally linked with metabolic comorbidities such as insulin resistance, hepatic steatosis, and dyslipidemia. However, the clinical impact of HCV eradication achieved by direct-acting antivirals (DAAs) on glucose and lipid homeostasis is still controversial. The study aimed to prospectively investigate whether antiviral therapy of HCV with DAAs alters glucose and lipid parameters. Methods: 50 patients with chronic HCV who were treated with DAAs were screened, and 49 were enrolled in the study. Biochemical and virological data, as well as noninvasive liver fibrosis parameters, were prospectively collected at baseline, at the end of treatment (EOT) and 12 and 24 weeks post-treatment. Results: 45 of 46 patients achieved sustained virologic response (SVR). The prevalence of insulin resistance (HOMA-IR) after HCV clearance was significantly lower, compared to baseline (5.3 ± 6.1 to 2.5 ± 1.9, *p* < 0.001), which is primarily attributable to a significant decrease of fasting insulin levels (18.9 ± 17.3 to 11.7 ± 8.7; *p* = 0.002). In contrast to that, HCV eradication resulted in a significant increase in cholesterol levels (total cholesterol, low-density lipoprotein cholesterol (LDL-C), and high-density lipoprotein (HDL-C) levels) and Controlled Attenuated Score (CAP), although BMI did not significantly change over time (*p* = 0.95). Moreover, HOMA-IR correlated significantly with noninvasive liver fibrosis measurements at baseline und during follow-up (TE: *r* = 0.45; *p* = 0.003, pSWE: *r* = 0.35; *p* = 0.02, APRI: *r* = 0.44; *p* = 0.003, FIB-4: *r* = 0.41; *p* < 0.001). Conclusion: Viral eradication following DAA therapy may have beneficial effects on glucose homeostasis, whereas lipid profile seems to be worsened.

## 1. Introduction

Hepatitis C Virus (HCV) infection is a global public health burden, affecting approximately 71 million persons worldwide [1]. Chronic HCV (CHC) causes a chronic state of inflammation, thus increasing the risk for severe consequences such as end-stage liver disease, hepatocellular carcinoma, and liver-related mortality [2]. Beyond that, there is emerging evidence that CHC is causally linked to cardiometabolic risk factors such as insulin resistance (IR), hepatic steatosis, and hyperlipidemia [3,4,5]. Accordingly, patients with CHC are more likely to develop type 2 diabetes mellitus (T2DM), as compared to the general population or to patients with chronic viral hepatitis, other than HCV [6,7,8,9]. Although the precise molecular mechanisms are not fully understood, there is considerable evidence for several interactions between HCV and the insulin signaling pathway. Oxidative stress and upregulation of proinflammatory cytokines have been identified as key mechanisms in the pathogenesis of HCV-mediated IR [10,11].

The reduction of IR and T2DM during and after HCV eradication has been extensively examined within the literature, but the results are still conflicting. Several studies reported that SVR induced by interferon (IFN)-based or direct-acting antiviral (DAA) therapies has beneficial effects on the glycometabolic control [12,13,14,15]. However, a statistically significant effect could not always be detected, and there is still a lack of prospective long-term studies which confirm a maintaining effect of SVR on IR over time [16,17,18].

Beyond that, HCV alters lipid pathways in order to enhance its replication. Components of VLDL are especially involved in the process of HCV assembly and secretion [3]. Higher SVR rates have been reported by combining statins with IFN-based therapy, which further confirms the pathophysiological relevance of lipids in the HCV replication cycle [19,20,21]. However, changes in lipid parameters during and after the IFN-free DAA regimen have not been adequately assessed.

The aim of the present study was to prospectively evaluate the impact of HCV clearance following direct-acting antiviral agent (DAA) treatment on glucose and lipid homeostasis.

## 2. Material and Methods

### 2.1. Patients

All patients aged 18 and over with CHC who were referred to the outpatient clinic of the Department of Gastroenterology and Hepatology of the University of Frankfurt for antiviral treatment with DAA, from August 2018 to April 2019, were considered for enrollment in the study. Positive HCV RNA in plasma for at least six months proved the diagnosis of CHC.

Criteria of exclusion were co-infection with human immunodeficiency virus and other viral hepatitis, decompensated cirrhosis (Child ≥ B), and significant alcohol consumption (>20 g/d in women and >30 g/d in men). DAA-therapy was administered according to the European Association for the Study of the Liver guidelines for HCV treatment 2018 and 2019 [22]. Patient demographics, including age at inclusion, gender, ethnicity, and previous history of antiviral treatment, were recorded at baseline. BMI was assessed at baseline and throughout follow-up.

The study and its protocol were approved by the local ethics committee of Frankfurt University Hospital, in accordance with the Declaration of Helsinki (ethics committee reference number: 392/17). Patients provided written informed consent prior to study participation.

### 2.2. Serum Biomarkers

At baseline and at each follow-up visit, a biochemical dataset including liver and renal function tests, virological values, and biochemical parameters for calculating FibroTest (FT), Fibrosis 4 (FIB-4), and aspartate-aminotransferase-to-platelet ratio index (APRI) was assessed.

Beyond that, fasting samples were prospectively collected for measurements of lipids (serum total cholesterol, high-density lipoprotein (HDL), low-density lipoprotein (LDL), and triglycerides) and diabetic values (HbA1c, serum fasting glucose (FG), and serum insulin).

Subjects with HOMA-IR levels >3 were defined as IR, as previously described [23]. HOMA-IR was calculated according to the homeostatic assessment model: fasting glucose (mmol/L) × fasting glucose (mmol/L)/22.5.

FibroTest is a patented fibrosis marker which was developed by BioPredictive (Paris, France). It was calculated by using a combination of six biomarkers [24]. FIB-4 and APRI were calculated as previously described [25,26]. The thresholds used for each fibrosis marker to discriminate significant fibrosis and cirrhosis were those reported in the original publications [24,25,26].

### 2.3. Liver Stiffness Measurement

Liver stiffness was performed by ARFI imaging and Transient Elastography (TE) for each patient, under fasting conditions. Details of the examination procedure and technical principles of both methods have been described elsewhere [27]. The examinations were performed in the right lobe of the liver during real-time B-mode imaging.

Ten successful measurements were obtained in each patient. The median value was taken as representative. Test results for ARFI and TE were expressed in meters per seconds (m/s) and in kPa, respectively. The thresholds used for diagnosing significant fibrosis and cirrhosis were those described previously in the literature: 1.37 and 1.75 kPa for ARFI imaging, and 7.2 and 11.0 kPa for TE [27,28].

### 2.4. Statistical Analysis

Data were expressed as mean ± standard deviation, if normally distributed, and as median and interquartile-range (IQR), if not normally distributed. Associations between categorial variables were tested by Spearman’s correlation coefficients and their associated probability. Comparisons between groups were assessed by using the Mann–Whitney *U*-test. Friedman’s test was carried out to detect longitudinal differences of metabolic parameters and noninvasive fibrosis assessments during 24 weeks of follow-up.

A *p* value of less than 0.05 was judged to be statistically significant. All analyzes were performed by using IBM SPSS 26.0 statistical software package (SPSS/IBM, Munich, Germany).

## 3. Results

A total of 49 patients were enrolled. Three patients discontinued the study prematurely. A total of 46 consecutive patients (47.8% male; mean age 51.7 ± 14.7; age range: 23–82) continued at least until the end of treatment, and the metabolic effects of HCV eradication could be investigated. A summary of baseline patient demographics, biochemical, and metabolic features is listed in Table 1.

HCV genotypes were distributed as follows: 36.9% genotype 1a (17/46), 28.2% genotype 1b (13/46), 21.8% genotype 2 (10/46), 2.2% genotype 3 (1/46), and 10.9% genotypes 4–6 (5/46). Antiviral regimens for treatment of HCV infection were velpatasvir/sofosbuvir, with (1/46) or without ribavirin (5/46), glecaprevir/pibrentasvir (36/46), elbasvir/grazoprevir (3/46), and sofosbuvir/velpatasvir/voxilaprevir (1/46).

Out of 46 patients, 13 (28.2%) were cirrhotic (all Child Pugh A), 2 (4.3%) had advanced fibrosis (F3), 10 (21.7%) had moderate fibrosis (F2), and 21 (45.8%) had no significant fibrosis (F0–1).

BMI was 25.4 ± 4.2, reflecting a study population in the upper range of normal weight. At baseline, mean fasting plasma glucose (FG) levels were in the prediabetes range (105.0 ± 37.7). Mean fasting plasma insulin was 21.7 ± 27.9 (HOMA-IR: 6.0 ± 9.1). IR, defined by HOMA-IR > 3, was present in 25 patients (54.3%). Twenty-one patients (45.6%) had normal HOMA-IR values (<3). Patients with IR were significantly older (*p* = 0.03) and had significantly higher transaminases (AST: *p* = 0.02; ALT: *p* = 0.05), lipid parameters (total cholesterin: *p* = 0.01, LDL: *p* = 0.01; triglycerides: *p* = 0.02) and noninvasive liver fibrosis values (TE: *p* = 0.008; pSWE: *p* = 0.02; APRI: *p* = 0.01; FIB-4: *p* = 0.004; FT: *p* = 0.007).

IR was significantly more common in patients with cirrhosis than in related controls (58.6% vs. 41.3%; *p* = 0.02). At baseline and at single follow-ups there was a statistically significant positive correlation of HOMA-IR levels with noninvasive fibrosis assessments (baseline: TE: Spearman’s rho = 0.46; *p* = 0.003, pSWE: *r* = 0.35; *p* = 0.02, APRI: *r* = 0.41; *p* = 0.005, FIB-4: *r* = 0.44; *p* = 0.003; FT: *r* = 0.5; *p* < 0.001; Table S1) and liver parameters (baseline: AST: *r* = 0.4; *p* = 0.006, ALT: *r* = 0.31; *p* = 0.04, GGT: *r* = 0.55, *p* < 0.001; Table S1). Significant inverse correlations could be detected between HOMA-IR and lipid parameters (baseline total cholesterin: *r* = −0.36, *p* = 0.02; LDL: *r* = −0.43, *p* = 0.003). Mean total and LDL cholesterol were 158.1 ± 42.4 and 94.8 ± 34.7, respectively, indicating average values below the normal range. Mean HDL and triglyceride levels at baseline were 49.8 ± 12.7 and 112.7 ± 73.6, respectively.

### Longitudinal Study

Forty-six patients continued a full course of treatment (EOT). After that, two patients were lost before week 12 of follow-up, and three patients before week 24 of follow-up. SVR was detected in 43 patients. One patient experienced a relapse at 12 weeks of post-DAA treatment.

Changes in liver parameters, HCV RNA, and liver fibrosis parameters relative to baseline are presented in Table 2.

**Figure 1:**
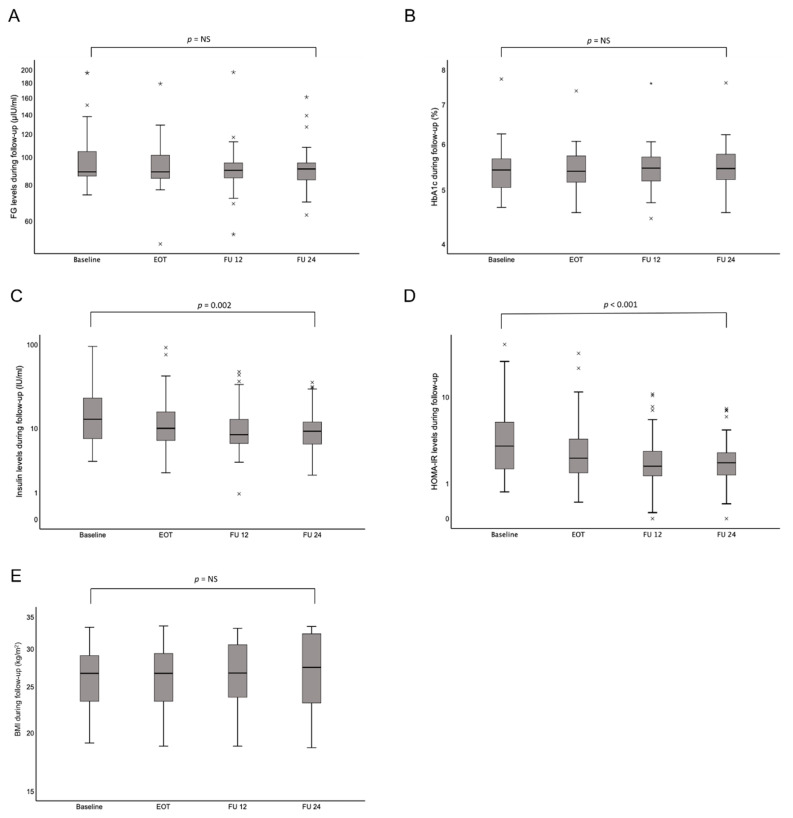
Changes in metabolic parameters over time, during and after DAA therapy. (**A**) Fasting glucose (FG), (**B**) HbA1c, (**C**) insulin, (**D**) HOMA-IR, and (**E**) BMI. The top and bottom of the boxes are the first and third quartiles, respectively. The middle line through the middle of each box represents the median. × represents mild outliers which lie between 1.5 and 3.0 times the IQR. * represents extreme outliers which lie more than 3.0 times the IQR. Abbreviations: BMI, body mass index; FG, fasting glucose; HOMA-IR, homeostasis model of insulin resistance.

As expected, transaminase levels significantly reduced after viral clearance by DAA. Regarding noninvasive liver fibrosis assessments, we found decreases in TE (*p* = 0.01), pSWE (*p* < 0.001), APRI (*p* < 0.001), FIB-4 (*p* = 0.12) and FibroTest values (*p* < 0.001; Table 2). Conversely, a significant increase in CAP values (*p* = 0.03; Table 2) was observed at the end of follow-up relative to baseline. Table 3 shows metabolic parameters at baseline and at each follow-up visit. FG and HbA1c did not significantly change over time, although they tended to decrease from baseline to FU24 (FG: *p* = 0.11; HbA1c: *p* = 0.82; Figure 1A,B). However, a significant reduction over time could be detected in basal insulin levels and HOMA-IR (insulin: 18.9 ± 17.3 to 11.7 ± 8.7; *p* = 0.002; HOMA-IR: 5.3 ± 6.1 to 2.5 ± 1.9, *p* < 0.001; Table 3; Figure 1C,D). Accordingly, the proportion of patients with IR was significantly lower at et end of follow-up, compared to baseline: 24 weeks after EOT, nine patients (19.6%) were IR vs. 25 patients (54.3%) at baseline (*p* < 0.001). These changes in insulin sensitivity could be detected, although BMI did not significantly decrease over time (25.4 ± 4.2 to 26.3 ± 5.6; *p* = 0.95; Figure 1E).

**Figure 2:**
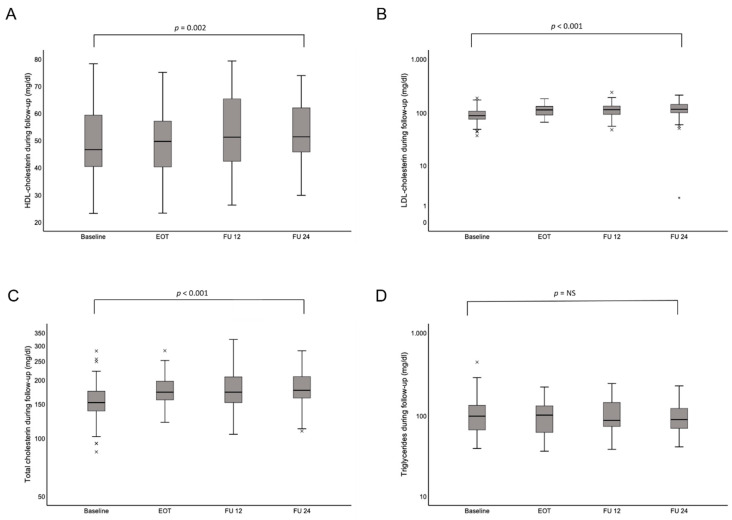
Changes in lipid parameters over time, during and after DAA therapy. (**A**) HDL-cholesterin, (**B**) LDL-cholesterin, (**C**) total cholesterin, and (**D**) triglycerides. The top and bottom of the boxes are the first and third quartiles, respectively. The middle line through the middle of each box represents the median. × represents mild outliers which lie between 1.5 and 3.0 times the IQR. * represents extreme outliers which lie more than 3.0 times the IQR. Abbreviations: EOT, end of treatment; FU12, 12 weeks; IQR, interquartile range.

Moreover, HCV eradication following DAA treatment affected the lipid profile. Serum HDL, LDL, and total cholesterol significantly increased at the end of follow-up (*p* = 0.002; *p* < 0.001; *p* < 0.001; Table 3; Figure 2A–C). However, we did not observe an increase in triglycerides, which were even slightly lower at the end of follow-up, compared to baseline (*p* = 0.83, Figure 2D).

The top and bottom of each box represent the first and third quartiles, respectively. The middle line represents the median. Abbreviations: EOT, end of treatment; FU12, 12 weeks.

## 4. Discussion

The association between chronic HCV infection and metabolic disease, involving IR, hepatic steatosis, and altered lipid homeostasis, has been extensively examined in the literature. However, there has still been insufficient evaluation of metabolic effects of interferon-free DAA treatment during therapy and after SVR.

The novelty of our study lies in a prospective comprehensive analysis of metabolic findings, several noninvasive liver fibrosis parameters, and the CAP score, a measure of steatosis, in individuals undergoing interferon-free DAA treatment.

In our study, based on a well-characterized cohort of patients with chronic HCV infection, SVR was found to be associated with a significant improvement in HOMA-IR, thus supporting theories of a direct pathophysiologic mechanism between T2DM and chronic HCV. Interestingly, a highly significant decrease in insulin levels primarily contributed to this reduction of HOMA-IR, whereas FG levels did not significantly change over time. As previously shown, IR in chronic HCV mainly results from an increased peripheral resistance to insulin activity which is triggered by proinflammatory cytokines and chronic hepatic inflammation [29,30]. Consequently, an improvement of hepatic inflammation during and after HCV eradication probably contributes to the reduction of insulin levels and consecutively of IR.

Of course, there are also other factors, besides viral infection, which have to be taken into consideration when interpreting these results. An improvement in health perception and physical self-awareness during HCV treatment, for example, may have a positive effect on a more active lifestyle. However, we did not detect a significant weight reduction in our cohort, which strengthens the suggestion that chronic HCV might be an independent risk factor for the development of IR [12,31].

T2DM has been proposed to promote the progression of hepatic inflammation and fibrosis in HCV-infected patients. Several studies have reported a significant association between T2DM and the incidence of liver fibrosis and cirrhosis in patients with CHC [32,33,34]. However, there are also negative study results regarding this issue, and the association of glycemic control and liver fibrosis has not been sufficiently examined until now [35,36]. In our study, IR was significantly more common in patients with cirrhosis, compared to related controls. Beyond that, a significantly positive correlation could be detected between IR and several noninvasive liver fibrosis parameters at baseline, during therapy, and after HCV eradication, which might propose a pathophysiological association between T2DM and hepatic fibrosis. However, care should be taken when interpreting these results, as biochemical response to HCV therapy may influence not only serum biomarkers but also liver stiffness measurements. Moreover, the rapid decline of all noninvasive fibrosis markers at an early time point (six months post-EOT) after therapy suggests that the results rather reflect the regression of necroinflammation and liver edema than the reversal of fibrosis. Accordingly, the overall correlation between IR and noninvasive fibrosis parameters at baseline and during follow-up might reflect the complex interaction between inflammatory cytokines, IR, and hepatic steatosis which promotes the progression of fibrosis during chronic HCV and which is resolved by antiviral therapy.

Beyond exploring the effects of HCV eradication on glycemic control, we also evaluated the lipid profile. In our study, we observed a significant increase in total, HDL, and LDL cholesterol during and after antiviral therapy. HCV is known to hijack lipid metabolic pathways for virion maturation and secretion. Very low density lipoproteins (VLDL) are essential for viral assembly and envelope acquisition, building complexes with circulating HCV, termed lipoviral particles, which contain host apolipoproteins (APOs) such as APOB, APOE, and APOC3 [3]. The formation of those lipoviral particles is thought to enable HCV to enter hepatocytes by binding LDL-receptors [37]. Accordingly, chronic HCV is associated with altered lipid metabolism in terms of enhanced lipogenesis and reduced secretion of lipoproteins, leading to lower concentrations of total cholesterol, HDL, and LDL. The rapid increase of lipid levels during and after HCV clearance likely reflects the reversal of lipid metabolism perturbation by inhibiting HCV replication.

Interestingly, a significant increase over time could also be observed for CAP, a measure of liver steatosis. This finding was obviously independent of weight gain, since no significant change in BMI could be detected over time. The increase of liver steatosis and lipid levels and lipid levels resulting from HCV eradication might suggest a direct causal relationship that needs to be further studied.

Our study has some limitations. It is limited to a relatively short-term follow-up. Therefore, further studies are required to investigate whether these observations are maintained over a more extended follow-up period. Beyond that, a possible confounder when analyzing and interpreting our results could be the heterogeneity of our study population, which consists of different viral genotypes and different stages of liver fibrosis. Patients suffering from advanced stages of cirrhosis are especially lacking in the present study.

## 5. Conclusions

In conclusion, our results suggest a significant and continuous reduction of liver stiffness by noninvasive fibrosis measurements after DAA-induced SVR. The decline during and after therapy to FU 12 rather reflects the reduction in necroinflammation activity than fibrosis regression during this short period of follow-up. Moreover, our data demonstrate that HCV eradication following DAA treatment leads to a progressive reduction of insulin resistance. Insulin resistance was significantly positively correlated with noninvasive fibrosis assessments throughout follow-up, implicating a pathophysiological link which might be relevant for the progression of fibrosis during chronic HCV.

Moreover, our results show a dynamic change of serum lipid parameters with a significant increase in lipid parameters and CAP values from baseline to follow-up. The impact of these findings on patients’ health should be investigated.

## Figures and Tables

**Table 1:** Baseline patient characteristics.

Characteristics	Patients (*n* = 46)
Patient age (years), mean ± SD	51.7 ± 14.1
Male gender, *n* (%)	22 (47.8%)
Caucasian ethnicity, *n* (%)	32 (69.0%)
BMI (kg/m^2^), mean ± SD	25.4 ± 4.2
HCV genotype 1a/1b/2/3/4–6, *n* (%)	17 (36.9)/13 (28.2)/10 (21.7)/1 (3)/5 (10.8)
Median baseline HCV RNA (log_10_, IU/mL)	6.06
Previous antiviral therapy, *n* (%)	6 (13.0)
Metabolic Comorbidity, *n* (%)	
Hyperlipidemia	5 (8.6%)
Hypertension	13 (28.3%)
Diabetes mellitus	5 (10.9%)
Compensated cirrhosis, *n* (%)	12 (26.1%)
Child Class A/B, *n* (%) *	12(26.1)/0(0)
MELD score, mean ± SD *	9.7 ± 4.0
Total bilirubin (mg/dL), mean ± SD	0.54 ± 0.3
Serum albumin (g/dL), mean ± SD	4.4 ± 0.4
AST (IU/L), mean ± SD	57.6 ± 39.6
ALT (IU/L), mean ± SD	73.9 ± 53.3
GGT (IU/L), mean ± SD	79.1 ± 68.9
Total cholesterol (mg/dL), mean ± SD	158.1 ± 42.4
LDL cholesterol (mg/dL), mean ± SD	94.8 ± 34.7
HDL cholesterol (mg/dL), mean ± SD	49.8 ± 12.7
Triglycerides (mg/dL), mean ± SD	112.7 ± 73.6
HbA1c (%), mean ± SD	5.4 ± 0.5
Fasting glucose (mg/dL), mean ± SD	105.0 ± 37.7
Insulin (µIU/mL), mean ± SD	18.9 ± 17.3
HOMA-IR, mean ± SD	5.3 ± 6.1

ALT, alanine aminotransferase; AST, aspartate aminotransferase; BMI, body mass index; GGT, gamma glutamyl transpeptidase; HCV, hepatitis C virus; HDL, high-density lipoprotein; LDL, low-density lipoprotein; MELD, model for end-stage liver disease. * Patients with cirrhosis.

**Table 2:** Liver parameters, HCV RNA, and liver stiffness during follow-up.

Variable	Baseline (*n* = 46)	EOT (*n* = 46)	FU12 (*n* = 44)	FU24 (*n* = 41)	*p* *
AST (IU/L), mean ± SD	57.6 ± 39.6	28.6 ± 15.8	27.6 ± 12.1	27.4 ± 14.0	<0.001
ALT (IU/L), mean ± SD	73.9 ± 53.3	23.4 ± 13.8	23.9 ± 20.9	24.2 ± 17.9	<0.001
GGT (IU/L), mean ± SD	79.1 ± 68.9	175.5 ± 32.8	28.0 ± 30.9	29.0 ± 29.6	<0.001
Total bilirubin (IU/L), mean ± SD	0.5 ± 0.3	0.6 ± 0.4	0.5 ± 0.3	0.6 ± 0.4	0.14
Serum albumin (g/dL), mean ± SD	4.4 ± 0.4	4.4 ± 0.4	4.5 ± 0.3	4.6 ± 0.3	0.09
Median HCV RNA (log_10_, IU/mL)	6.1	Undetectable	Undetectable	Undetectable	<0.001
Median TE (kPa)	7.7	5.8	5.3	5.4	0.01
Median ARFI (m/s)	1.4	1.4	1.1	1.1	<0.001
Median APRI score	0.6	0.3	0.3	0.3	<0.001
Median FIB-4 score	1.5	1.1	1.2	1.1	0.11
Median FT score	1.4	0.5	0.7	0.3	<0.001
Median CAP (dB/M)	240.0	240.5	248.2	250.0	0.03

AST, aspartate aminotransaminase; ALT, alanine aminotransaminase; BMI, body mass index; CAP, Controlled Attenuated Score; FT, FibroTest; GGT, gamma-glutamyl transpeptidase; SD, standard deviation. *p* *, Friedman Test for comparison of baseline with FU24, *n* = 41.

**Table 3:** Changes in metabolic parameters during and after DAA therapy.

Variable	Baseline (*n* = 46)	12W (*n* = 46)	24W (*n* = 44)	48W (*n* = 41)	*p* *
Fasting glucose (mg/dL); mean ± SD	105.0 ± 37.7	105 ± 37.7	94.5 ± 23.8	93.1 ± 17.5	0.11
HbA1c (%); mean ± SD	5.4 ± 0.5	5.4 ± 0.6	5.6 ± 0.6	5.5 ± 0.6	0.82
Insulin (µIU/mL); mean ± SD	18.9 ± 17.3	16.5 ± 18.5	12.2 ± 11.3	11.7 ± 8.7	0.001
HOMA-IR; mean ± SD	5.3 ± 6.1	3.9 ± 5.0	3.9 ± 5.0	2.5 ± 1.9	0.001
BMI (kg/m^2^); mean ± SD	25.4 ± 4.2	25.1 ± 5.6	25.1 ± 4.1	26.3 ± 5.6	0.95
HDL cholesterol (mg/dL); mean ± SD	49.8 ± 12.7	49.4 ± 11.1	52.5 ± 13.1	53.8 ± 12.4	0.002
LDL cholesterol (mg/dL); mean ± SD	94.8 ± 34.2	111.8 ± 33.7	116.5 ± 37.1	120.5 ± 39.8	<0.001
Total cholesterol (mg/dL); mean ± SD	158 ± 42.4	175.5 ± 32.8	176.7 ± 43.3	182.6 ± 37.7	<0.001
Triglycerides (mg/dL); mean ± SD	112.7 ± 73.6	104.3 ± 47.2	103.6 ± 50.3	101.3 ± 45.1	0.83

AST, aspartate aminotransaminase; ALT, alanine aminotransaminase; BMI, body mass index; GGT, gamma-glutamyl transpeptidase; SD, standard deviation; *p* *, Friedman Test for comparison of baseline with FU24, *n* = 41.

## Supplementary Materials

The following are available online at https://www.mdpi.com/2077-0383/9/9/2702/s1. Table S1: Correlation of HOMA-IR with noninvasive liver fibrosis assessments and liver parameters, at different time points, during and after antiviral treatment.
