# Supplementary material for: Hepatitis C Clearance by Direct-Acting Antivirals Impacts Glucose and Lipid Homeostasis
Source: J Clin Med. 2020 Aug 21;9(9):2702. doi: 10.3390/jcm9092702 (PMC7564474; doi:10.3390/jcm9092702)
Supplement: Supplementary file 1 [file jcm-09-02702-s001.pdf]

Appendix

Table 1. Correlation of HOMA-IR with non-invasive liver fibrosis assessments and liver parameters at different time points during and after antiviral treatment

Variables	HOMA-IR, Baseline, n=46		HOMA-IR, EOT, n=44		HOMA-IR, FU12, n=44		HOMA-IR, FU24, n=41	
TE (kPa)	r=0.46	p=0.003	r=0.43	p=0.009	r=0.24	p=0.04	r=0.56	p=0.03
pSWE (m/s)	r=0.35	p=0.02	r=0.11	p=0.49	r=0.10	p=0.52	r=0.20	p=0.23
FIB-4	r=0.44	p=0.003	r=0.23	p=0.12	r=0.21	p=0.19	r=0.21	p=0.19
APRI	r=0.41	p=0.005	r=0.36	p=0.02	r=0.37	p=0.01	r=0.28	p=0.04
FT	r=0.5	p<0.001	r=0.36	p=0.01	r=0.36	p=0.01	r=0.11	p=0.48
AST	r=0.4	p=0.006	r=0.39	p=0.007	r=0.35	p=0.01	r=0.23	p=0.16
ALT	r=0.31	p=0.04	r=0.45	p=0.002	r=0.33	p=0.03	r=0.44	p=0.05
GGT	r=0.55	pr<0.001	r=0.46	p=0.001	r=0.28	p=0.06	r=0.33	p=0.04

APRI, aspartate to platelet ratio index; FIB-4, fibrosis index based on four factors; EOT, end of treatment, FT, FibroTest; FU, follow-up; pSWE, point-shear wave elastography; TE, transient elastography.
